# RIPK3’s shape-shifting scaffold: how kinase domain conformation fine-tunes necroptosis

**DOI:** 10.1038/s41418-026-01678-w

**Published:** 2026-02-10

**Authors:** Daniela Ungureanu

**Affiliations:** 1 Disease Networks Unit, Faculty of Biochemistry and Molecular Medicine, University of Oulu, Oulu, Finland; 2 Institute for Molecular Medicine Finland (FIMM), Helsinki Institute of Life Science (HiLIFE), University of Helsinki, Helsinki, Finland

**Keywords:** Experimental models of disease, Protein folding

Necroptosis is a tightly regulated form of cell death that culminates in membrane rupture and the release of inflammatory damage-associated molecular patterns [1]. Once considered merely cellular accidents, programmed necrotic death pathways have emerged as sophisticated biological mechanisms with ancestral roots in host defence against pathogens [2]. The molecular execution of necroptosis is orchestrated by receptor-interacting serine/threonine-protein kinase 3 (RIPK3), which phosphorylates and activates the pseudokinase mixed lineage kinase domain-like protein (MLKL). Upon RIPK3-mediated phosphorylation within its pseudokinase domain activation loop, MLKL undergoes conformational changes that promote its oligomerization, translocation to the plasma membrane, and insertion into the lipid bilayer that culminates in membrane permeabilization and cell lysis [3]. In an elegant study now published in *Cell Death & Differentiation*, Chiou et al. challenge the prevailing view of RIPK3 as solely a catalytic activator of MLKL [4]. Their findings reveal that the three-dimensional conformation of RIPK3’s kinase domain, independent of its enzymatic activity, functions as a molecular rheostat that dynamically tunes cellular commitment between necroptotic and apoptotic death pathways.

The identification of MLKL as the terminal effector of necroptosis marked a turning point in the study of regulated cell death [5]. MLKL is a pseudokinase, a protein that has lost the catalytic activity through evolution, yet retained its capacity to regulate signalling [6]. Its discovery revealed that pseudokinases can function not just as inert remnants but as dynamic molecular switches. In the necroptotic pathway, MLKL transforms from a dormant monomer into an oligomeric, membrane-disrupting killer once activated by its upstream partner, RIPK3 [7].

RIPK3 has traditionally been viewed primarily through the lens of its catalytic function, leading to MLKL phosphorylation to trigger necroptosis. This view was supported by early observations that RIPK3-deficient mice are viable and phenotypically normal under basal conditions, suggesting that RIPK3's primary role is executing necroptotic death in response to specific signalling cues [8]. However, the work from Chiou et al. upends this simple narrative, revealing that RIPK3 functions as a sophisticated molecular scaffold whose conformation dictates cell fate decisions.

**Fig. 1:**
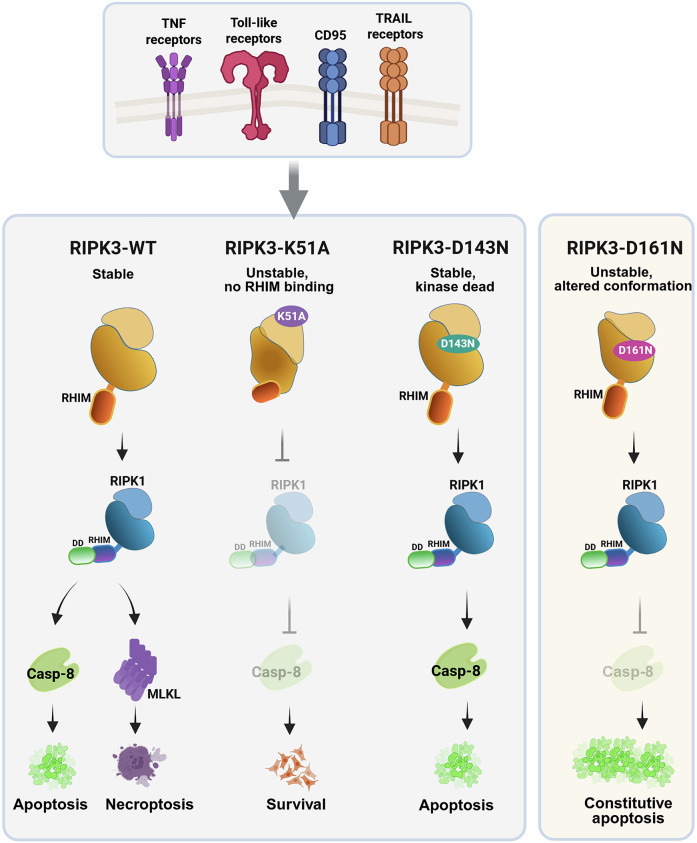
RIPK3 kinase domain conformation tunes scaffolding function and cell fate decisions downstream of death receptor signalling. Top panel: Necroptosis can be initiated upon engagement of death receptors (TNF receptors, Toll-like receptors, CD95, TRAIL receptors) at the plasma membrane, triggering assembly of intracellular death-signalling complexes. Bottom panel: Schematic representation of how RIPK3 kinase domain stability and conformation control assembly of death-signalling complexes and downstream cell fate outcomes. RIPK3-WT (stable): Wild-type RIPK3 (gold, with RHIM domain shown in orange) forms a stable complex with RIPK1 through RHIM-RHIM interactions. RIPK1’s death domain (DD, green) recruits Caspase-8, enabling stimulus-dependent apoptosis. Concurrently, active RIPK3 kinase phosphorylates MLKL (purple), triggering oligomerization, membrane translocation, and necroptotic cell death. RIPK3-K51A (unstable): The K51A mutation renders RIPK3 conformationally unstable and unable to engage RIPK1 via RHIM domains, preventing assembly of death-signalling complexes and promoting cell survival. RIPK3-D143N (stable, kinase dead): The D143N mutation impairs the catalytic activity while maintaining wild-type-like protein stability and conformation. RIPK3-D143N forms the complete RIPK3-RIPK1-Caspase-8 complex through preserved. RHIM and DD interactions. However, loss of kinase activity prevents MLKL phosphorylation and blocks necroptosis, while permitting Caspase-8-mediated apoptosis. RIPK3-D161N (unstable, altered conformation): The D161N mutation destabilizes RIPK3 and induces analtered kinase domain conformation. RIPK3-D161N retains the ability to bind RIPK1 via RHIM interactions, but the conformational perturbation impairs stable Caspase-8 recruitment to the complex. Despite this, RIPK3-D161N triggers constitutive apoptosis even in the absence of death-stimuli, through an incompletely understood mechanism. These findings demonstrate that RIPK3 kinase domain conformation, independent of catalytic activity, allosterically regulates the assembly, stoichiometry, and signalling output of death-inducing protein complexes. RIPK Receptor-Interacting serine/threonine-protein Kinase, RHIM RIP Homotypic Interaction Motif, DD death domain, MLKL mixed lineage kinase domain-like, TNF tumor necrosis factor, TRAIL TNF-related apoptosis-inducing ligand. This figure was created using Biorender.

The critical insight came from comparing three different kinase-inactivating mutations in RIPK3: K51A (ATP-binding site), D143N (catalytic loop), and D161N (activation loop). While all three mutations abolish RIPK3's ability to phosphorylate MLKL and execute necroptosis, they produce strikingly different cellular phenotypes. The RIPK3-K51A mutant was unstable and failed to bind either RIPK1 or Caspase-8, rendering it essentially non-functional in death signalling. The RIPK3-D161N mutant, despite being unstable, retained RIPK1 binding but lost Caspase-8 interaction. Remarkably, this mutant constitutively triggered apoptosis even in the absence of external death stimuli. In stark contrast, RIPK3-D143N exhibited stability comparable to wild-type RIPK3 and preserved interactions with both RIPK1 and Caspase-8 following necroptotic stimulation (Fig. 1).

These observations led authors to propose a revolutionary model: the RIPK3 kinase domain functions as an allosteric regulator of protein-protein interactions, with different conformational states dictating distinct cell death outcomes. Using cellular thermal stability assays, they demonstrated that RIPK3-D143N maintains the structural integrity of wild-type RIPK3, while RIPK3-K51A and RIPK3-D161N are conformationally labile. This structural instability directly impacts RIPK3 scaffolding capacity.

The practical implications became clear when testing responses to various apoptotic stimuli. RIPK3-K51A failed to mediate TNF-induced apoptosis, while RIPK3-D143N supported this process comparably to wild-type protein. Conversely, when cells were treated with GSK843, a selective ATP-competitive RIPK3 inhibitor, RIPK3-K51A promoted apoptosis while RIPK3-D143N prevented it. These opposing behaviours reveal that different perturbations to RIPK3 kinase domain produce distinct conformational states, each with unique scaffolding properties that either promote or prevent the assembly of the apoptotic machinery. Co-immunoprecipitation experiments provided the molecular insights needed to explain these divergent outcomes. In the presence of caspase inhibitors and necroptotic stimuli, wild-type RIPK3 and RIPK3-D143N both recruited RIPK1 and Caspase-8 into a complex. RIPK3-K51A recruited neither protein, while RIPK3-D161N recruited RIPK1 but not Caspase-8. This suggests that the D161N mutation locks RIPK3 into a conformation that constitutively scaffolds RIPK1-mediated apoptotic signalling, explaining its lethal phenotype.

Given that RIPK3-D143N preserves wild-type stability and binding properties while ablating catalytic activity, the authors recognized it as an ideal mutant for dissecting RIPK3’s kinase- independent functions in vivo. They generated *Ripk3-D143N* knock-in mice using CRISPR/Cas9 editing and demonstrated that adult homozygous animals are viable, fertile, and phenotypically indistinguishable from wild-type littermates under basal conditions. The mutant protein is expressed at normal levels across all examined tissues and completely blocks TNF- induced necroptosis in fibroblasts and macrophages while permitting TNF-induced apoptosis, confirming that the D143N mutation specifically ablates necroptotic signalling without compromising other RIPK3 functions. However, *Ripk3-D143N* mice show a subtle but significant reduction in homozygote representation at weaning compared to Mendelian ratios at embryonic day 10.5, indicating partially penetrant embryonic lethality that mirrors observations with *Ripk3-D161N* and *Ripk3-S165D/T166E* strains, though far less severe. This suggests that even the conformationally stable D143N mutant experiences some liability during a critical perinatal checkpoint, highlighting the unique sensitivity of developmental processes to RIPK3 signalling.

The team validated the necroptosis-blocking capacity of RIPK3-D143N in two disease models. First, germline deletion of *Caspase-8* causes embryonic lethality due to unrestrained RIPK3-MLKL-dependent necroptosis, but *Casp8*-knockout mice are fully viable on the *Ripk3-D143N* background, definitively demonstrating that the D143N mutation prevents developmental necroptosis in vivo. Intriguingly, no *Casp8*-knockout mice heterozygous for *RIPK3-D143N* were observed at birth, indicating that one functional *RIPK3* allele combined with one functional *MLKL* allele is insufficient to protect against *Caspase-8* deficiency during development, a dosage sensitivity that underscores the tight balance between pro-death and pro-survival signals during embryogenesis.

Perhaps the most clinically relevant findings came from studying intestinal inflammation. Conditional deletion of *Caspase-8* from intestinal epithelial cells causes ileitis in mice, characterized by RIPK1-RIPK3-MLKL-dependent necroptosis and selective loss of Paneth cells. Crossing *Villin-Cre Casp8*-*floxed* mice onto the *Ripk3-D143N* background completely prevented Paneth cell loss, phenocopying the protection afforded by conditional *MLKL* deletion from intestinal epithelia. This demonstrates that cell-intrinsic necroptosis within epithelial cells, rather than infiltrating immune cells, drives Paneth cell death and subsequent inflammation, validating necroptosis as a therapeutic target in inflammatory bowel disease. Using an innovative immunohistochemistry protocol to detect phospho-activated RIPK3, the authors visualized necroptotic signalling at single-cell resolution. Surprisingly, only a small fraction of intestinal epithelial cells showed *phospho-RIPK3* positivity in *Caspase-8*-deficient ileum despite widespread Paneth cell loss, suggesting that *Caspase-8* deficiency alone is insufficient to trigger necroptosis and that an additional insult, perhaps microbial infection, metabolic stress, or barrier disruption, is required to activate *RIPK3* and execute Paneth cell death.

This study establishes a new paradigm for understanding RIPK3 function. Rather than simply serving as an on-off switch through phosphorylation, the RIPK3 kinase domain acts as a dynamic conformational sensor that integrates multiple inputs to tune its scaffolding capacity. Different conformational states allosterically regulate interactions with RIPK1 and Caspase-8, thereby controlling whether cells undergo necroptosis, apoptosis, or survive. This has important implications for RIPK3 inhibitor development, as the toxicity of ATP-competitive inhibitors like GSK843 likely reflects the specific conformation induced rather than an inevitable consequence of blocking catalytic activity. The D143N mutation demonstrates that kinase-dead RIPK3 can be well-tolerated if proper conformation is maintained, suggesting that next-generation RIPK3 inhibitors should stabilize benign conformations while blocking MLKL phosphorylation.
